# Progranulin promotes glioma progression via interaction with cathepsin D and serves as a diagnostic and prognostic biomarker

**DOI:** 10.1007/s12672-026-04529-9

**Published:** 2026-01-27

**Authors:** Chunming Zhao, Jiamei Guo, Zhong Zhou, Xulong Huang, Yifan Hai, Wenbo Gao, Chaohang Chen, Guokai Dong, Hongxing Cai, Shanshan Li

**Affiliations:** 1 Department of Human Anatomy, Xuzhou Medical University, Xuzhou, Jiangsu China; 2 Jiangsu Medical Engineering Research Center of Gene Detection, Xuzhou, Jiangsu China; 3 Judicial Expertise Office, Jiaxing University, Jiaxing, Zhejiang,, China; 4 Department of Forensic Medicine, Xuzhou Medical University, Xuzhou, Jiangsu China; 5 The First Clinical Medical College, Xuzhou Medical University, Xuzhou, Jiangsu China

**Keywords:** Cathepsin D, Glioma, Prognosis, Progranulins, Tumor biomarker

## Abstract

Progranulin (PGRN) plays a crucial role in the progression of various tumor types, making it a potentially valuable biomarker. However, the roles of PGRN vary across tumor types. Therefore, this study aimed to comprehensively analyze PGRN expression in different tumor types to better understand and apply biomarker-guided cancer therapy. The pan-cancer data were obtained from the University of California Santa Cruz Xena platform, and PGRN expression was extensively examined across various cancer types and the corresponding normal tissues. R packages were used for data analysis, including survival outcomes, baseline characteristic assessments, and univariate and multivariate Cox regression models to evaluate the prognostic significance of PGRN. Quantitative real-time polymerase chain reaction (qPCR), co-immunoprecipitation assay, and immunoblotting were performed for molecular interaction and expression. Real-time cell analysis and migration and invasion assays were conducted for phenotype examination. Results showed that PGRN promotes glioma cell proliferation, migration, and invasion. It also interacts with precursor cathepsin D (CTSD) and facilitates its maturation, thereby linking PGRN’s tumor-promoting effects to CTSD. PGRN can be used as a diagnostic and prognostic biomarker for gliomas. Our study offers a foundational framework for using PGRN in glioma diagnosis and prognosis. The potential of PGRN as both a diagnostic and prognostic biomarker was identified suggesting novel avenues for targeted therapeutic strategies in glioma management.

**Graphical abstract:**

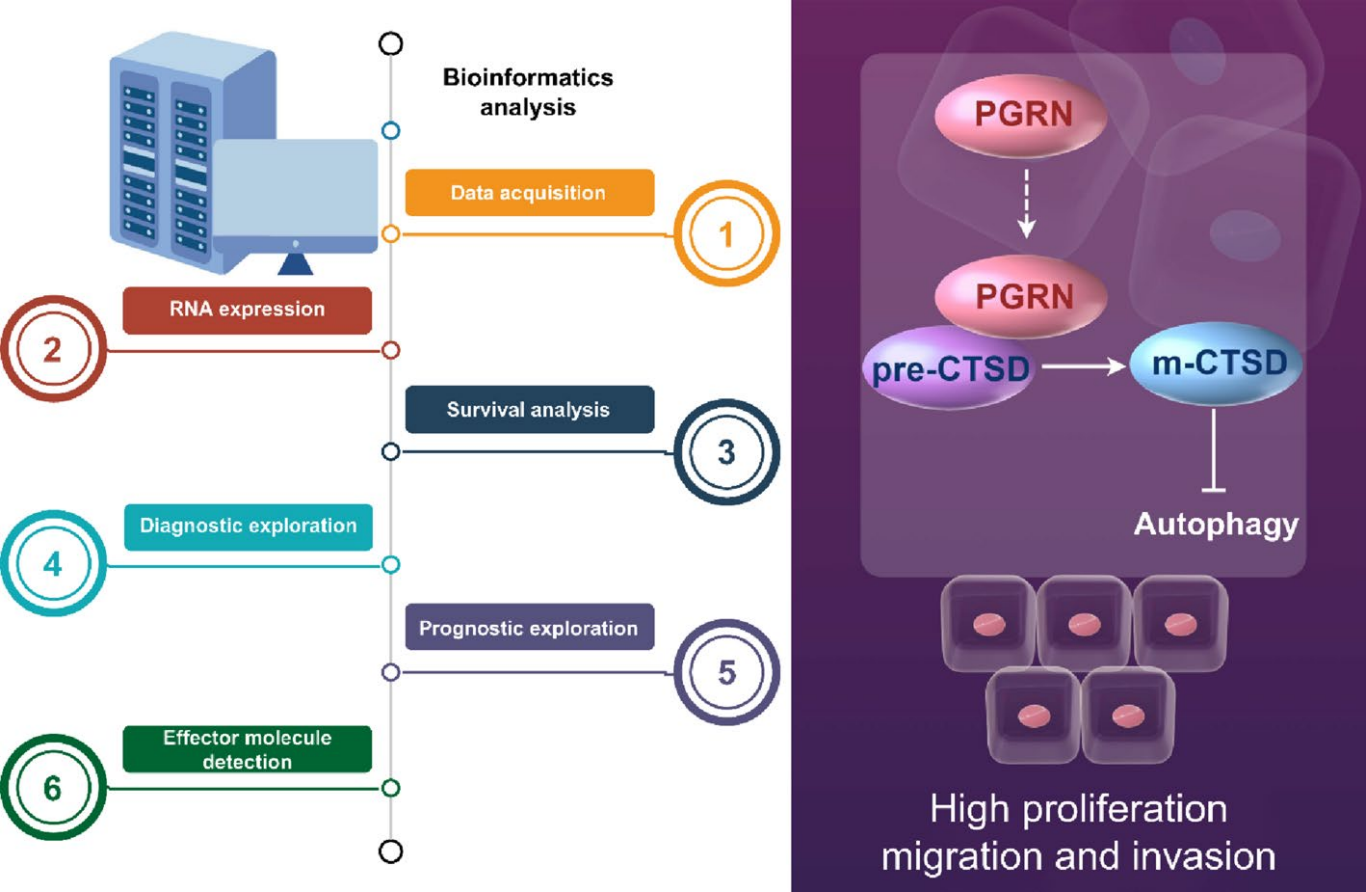

**Supplementary Information:**

The online version contains supplementary material available at 10.1007/s12672-026-04529-9.

## Introduction

Glioblastoma (GB), the most aggressive primary malignant brain tumor in adults, presents a dismal prognosis with a median survival of approximately 15–18 months despite advances in multimodal therapy [1–2]. The intricate pathogenesis of glioma, characterized by molecular heterogeneity, therapeutic resistance, and dynamic tumor microenvironment, underscores the critical need for reliable biomarkers to guide diagnosis, prognosis, and therapeutic strategies [3–4].

Progranulin (PGRN), a multifunctional growth factor, has garnered significant attention for its diverse roles in physiological and pathological processes, including inflammation, cell proliferation, and neural differentiation [5–7]. Within the central nervous system (CNS), PGRN demonstrates critical involvement in neuroinflammation, neurodegeneration, cerebral ischemia, traumatic brain injury, and glioma stemness [8–12]. Notably, PGRN exhibits minimal expression in normal brain tissue but significant upregulation in glioma specimens, particularly in tumor-associated vasculature and infiltrative zones, correlating with poor patient outcomes [10].

Emerging evidence highlights PGRN’s multifaceted role in GB pathogenesis. It contributes substantially to treatment resistance, particularly by enhancing temozolomide chemoresistance through regulation of DNA damage repair pathways and maintenance of tumor stemness [11]. Recent investigations have further revealed that PGRN facilitates immunosuppression within the glioma microenvironment by polarizing macrophages toward an M2-like phenotype and upregulating immune checkpoint molecules [13]. Some research has also identified novel aspects of PGRN’s function in glioma progression. Chen et al. demonstrated that PGRN mediates metabolic reprogramming through regulation of mitochondrial function, indicating a new mechanism for its role in treatment resistance [14]. These findings substantially expand our understanding of PGRN’s multifaceted role in glioma pathogenesis.

This study aimed to perform a comprehensive pan-cancer analysis of PGRN, with particular emphasis on its diagnostic and prognostic significance in glioma. By integrating expression profiling, survival analysis, and clinical correlation data across human malignancies, we sought to validate PGRN’s potential as a valuable biomarker for improving glioma management and guiding future therapeutic development.

## Methods

### Data acquisition

The data used for bioinformatics analysis were sourced from the datasets stored in the Xiantao Academic Platform (https://www.xiantaozi.com/) and the Gene Expression Profiling Interactive Analysis (GEPIA) platform (http://gepia2.cancer-pku.cn/#index). For the Xiantao Academic Platform, the Cancer Genome Atlas (TCGA; RRID: SCR_003193) and Genotype-Tissue Expression Program (GTEx; RRID: SCR_013042) datasets were obtained from University of California at Santa Cruz (CA, USA; RRID: SCR_011624) UCSC Xena (RRID: SCR_018938, https://xenabrowser.net/datapages/). TCGA provides comprehensive genomic data for various cancers, while GTEx offers expression data for normal tissues across multiple organs. Clinical data for the respective patients were acquired from the TCGA database (https://portal.gdc.cancer.gov). The prognostic data were obtained from a previous report [15].

### Diagnostic and prognostic value exploration

PGRN expression and pan-cancer survival analyses were performed using Gene Expression Profiling Interactive Analysis (GEPIA, RRID: SCR_026154) 2.0 [16] with default settings. GEPIA 2.0 processed and normalized RNA sequencing expression data from 9,736 tumors and 8,587 normal samples using a standardized pipeline. Baseline data for glioma (lower grade glioma [LGG] and GB) and liver hepatocellular carcinoma (LIHC) were analyzed using Xiantao Academic Platform with default settings. Specifically, RNA sequencing data from the TCGA-GB and TCGA-LGG projects were utilized by Xiantao Academic Platform and analyzed using the STAR (RRID: SCR_004463) pipeline, presented in trusted platform module format, and log2(value + 1) transformed. Samples lacking clinical data and normal tissues were excluded. Groups were classified as ‘high’ or ‘low’ PGRN expression based on the median threshold for each tumor type.

We assessed the diagnostic and prognostic significance of PGRN in various cancers. Receiver operating characteristic (ROC) analysis, prognostic nomogram, and univariate/ multivariate analysis OS analyses (Cox proportional hazards model) were performed using the Xiantao Academic Platform with default settings. Prognostic data were sourced from a publication by Liu et al. [15].

### Effector molecule detection

The top 100 genes associated with PGRN were identified using the GEPIA 2.0 platform (http://gepia2.cancer-pku.cn/#index), with results visualized by the R package circlize (RRID: SCR_002141, v0.4.1) in Xiantao Academic Platform. Subsequently, Gene Ontology (GO) and Kyoto Encyclopedia of Genes and Genomes (KEGG, RRID: SCR_012773) analyses were performed using clusterProfiler (RRID: SCR_016884, 4.4.4), with visualizations created by ggplot2 in Xiantao Academic Platform.

The immune infiltration score was determined via single-sample gene set enrichment analysis (ssGSEA) using the R package GSVA (RRID: SCR_021058 (4.2.1) [1.46.0]) in the Xiantao Academic Platform [17–18]. The top 100 protein–protein interaction networks of PGRN were constructed using STRING (RRID: SCR_005223, https://cn.string-db.org/) and visualized using the R packages igraph (RRID: SCR_019225 [1.4.1]) and ggraph (RRID: SCR_021239 [2.1.0]) in the Xiantao Academic Platform. Venn analysis of related and interacting genes was performed using ggplot2 [3.3.6] and VennDiagram (RRID: SCR_002414 [1.7.3]) in the Xiantao Academic Platform, which identified effector molecules. The expression of the target gene Cathepsin D (CTSD) was analyzed using ggplot2 [3.3.6], stats [4.2.1], and Companion to Applied Regression (RRID: SCR_022137 [3.1-0]) in the Xiantao Academic Platform. Correlation analysis between PGRN and CTSD was performed using GEPIA 2.0 (http://gepia2.cancer-pku.cn/#index).

### Cell culture

The GL261 mouse glioma cell line (CL0105, RRID: CVCL_X986) was obtained from Fenghui Biotechnology Co., Ltd. (Hunan, China). These cells were maintained in Dulbecco’s Modified Eagle Medium (DMEM; KeyGEN BioTECH, Nanjing, China) with 10% fetal bovine serum (FBS) at 37 °C in a 5% CO_2_ environment according to a previous report [19]. To overexpress PGRN, GL261 cells were infected with polybrene (Solarbio, Beijing, China) and a lentivirus containing either a negative control (NC) or full-length PGRN gene (Fenghui Biotechnology), followed by selection with puromycin (Solarbio) [20]. For CTSD knockdown, cells were transfected with Lipofectamine™ 2000 (Thermo Fisher Scientific, Shanghai, China) [21] and either non-targeting shRNA (NC) or CTSD-specific shRNA (Sangon Biotech, Shanghai, China).

### RNA isolation and quantitative real-time polymerase chain reaction (qPCR)

Total RNA was extracted using TRIzol reagent (Tiangen Biotech, Beijing, China), followed by reverse transcription to synthesize complementary DNA (cDNA) using a cDNA Reverse Transcription Kit (Tiangen Biotech). Moreover, qPCR was performed using Universal SYBR Green Mix (ABclonal, Wuhan, China) on an ABI 7500 Real-Time PCR system (Applied Biosystems, Thermo Fisher Scientific, Inc.) [22]. Data were analyzed using the ΔΔCt method. Specific primers used are listed in Online Resource 1: Table S1.

### Protein preparation and immunoblotting

Total protein was extracted from GL261 cells by western blotting and immunoprecipitation (IP) using a cell lysate from Beyotime Institute of Biotechnology (Shanghai, China), supplemented with 1% protease inhibitor (Vicmed, Xuzhou, China). Protein quantification was performed using the bicinchoninic acid assay (Beyotime Institute of Biotechnology). After quantification, protein samples were resolved by sodium dodecyl sulphate-polyacrylamide gel electrophoresis and transferred to polyvinylidene fluoride or polyvinylidene difluoride membranes. Membranes were blocked with 5% skim milk and incubated with specific antibodies [23]. Protein detection was performed using enhanced chemiluminescence reagents (ANALYTIK JENA AG, Jena, Germany). Antibody details are provided in Online Resource 1: Table S2.

### Co-immunoprecipitation (co-IP) assay

Protein lysates were incubated overnight at 4 °C with mouse IgG (Bioworld, Nanjing, Jiangsu, China) or PGRN antibody (Abcam, Cambridge, UK). Protein A/G agarose beads (Santa Cruz Biotechnology, CA, Dallas, TX, USA) were added for antibody immobilization and incubated overnight at 4 °C. After elution, proteins were analyzed by western blotting using PGRN and CTSD antibodies [24].

### Real-time cell analysis (RTCA)

RTCA was performed using the xCELLigence platform (ACEA Biosciences, San Diego, CA, USA). A total of 5 × 10³ pretreated cells were inoculated into E-16 plates and linked to the system within the incubator. Following a 30-min pre-incubation, the cell index was recorded at 15-min intervals over 72 h. All measurements were performed in triplicate for reliability [25].

### Cell migration and invasion assay

Cell migration and invasion were evaluated using Transwell assays. For migration, GL261 cells (1 × 10^5^ in 200 µL of serum-free DMEM) were introduced into a cell culture chamber in a 24-well plate (NEST; Wuxi, Jiangsu, China), with each well supplemented with 800 µL of DMEM containing 10% FBS. After 24 h, cells adhering to the outer filter were fixed and stained with 0.1% crystal violet (Vicmed). Microscopic imaging was performed using a Nikon ECLIPSE Ti microscope (Nikon, Tokyo, Japan) at 400× magnification. The invasion assay was performed similarly, with the upper chamber pre-coated with Matrigel (ABW, Shanghai, China), diluted in pre-cold serum-free medium to 1:8, and 60 µL applied per chamber [26]. Images were analyzed using ImageJ software (NIH, Bethesda, MD, USA) to quantify cell counts in each high-power field, with each point representing a count from an individual image.

### Statistical analysis

Statistical analyses and graphics were performed using R (4.2.1) in Xiantao Academic Platform and GraphPad Prism 9 (GraphPad Software, Boston, MA, USA). The Chi-square and Fisher’s exact tests were applied to baseline data, the Wilcoxon rank-sum test to differential expression, and Spearman’s correlation to immune infiltration scores. Experimental data (repeated > 3 times) are presented as means ± standard deviations. Differences were evaluated using analysis of variance, followed by multiple comparison tests. The level of significance was set at *p* < 0.05.

## Results

### PGRN expression in multiple cancers

GEPIA 2.0 assessed the mRNA expression of PGRN across 33 tumor types and matched normal tissues. The analysis revealed PGRN overexpression in cervical cancer, large B-cell lymphoma, GB, kidney papillary cell carcinoma, LGG, LIHC, ovarian cancer (OV), pancreatic cancer, melanoma, stomach cancer, testicular cancer, endometrioid cancer, and uterine carcinosarcoma than normal tissues (Online Resource 2: Fig. S1).

### Survival analysis

**Fig. 1:**
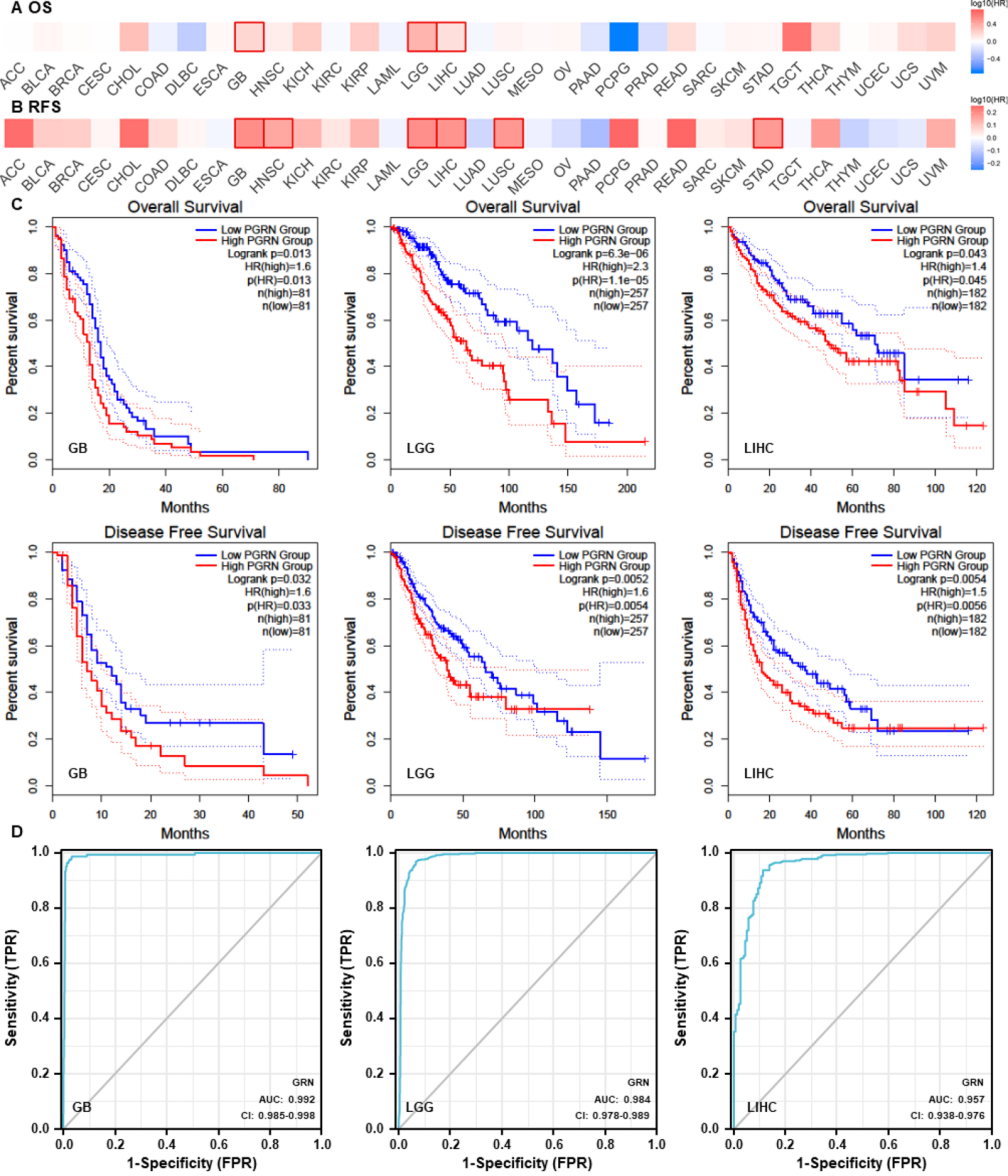
Prognostic and diagnostic value of PGRN expression in glioma and LIHC. **a**,** b** The survival analysis of the impact of PGRN on OS and (RFS) among patients with pan-cancer. **c** The Kaplan–Meier curves for OS and RFS of GB (left), LGG (middle), and LIHC (right), with patients showing significantly worse OS and RFS with high PGRN expression. Groups are divided based on the median threshold of GRN expression for each tumor type. Samples with GRN expression above the median were classified as ‘high’, while those below were classified as ‘low’. **d** ROC analysis reveals the diagnostic potential of PGRN expression in GB (left), LGG (middle), and LIHC (right). All survival analyses were performed using log-rank tests with significance indicated as *p* values. AUC values represent the area under the ROC curve quantifying diagnostic accuracy. PGRN (Progranulin), GRN (gene name of PGRN), OS (Overall Survival), RFS (recurrence-free survival), ROC (receiver operating characteristic), glioblastoma (GB), lower grade glioma (LGG), liver hepatocellular carcinoma (LIHC)

Survival analysis for OS and recurrence-free survival (RFS) was performed using the GEPIA 2.0 platform. The resulting survival maps revealed that individuals with elevated PGRN levels had significantly reduced survival rates in GB, LGG, and LIHC. A comparable pattern was observed for RFS among patients with GB, head and neck squamous cell carcinoma, LGG, LIHC, lung squamous cell carcinoma, and stomach adenocarcinoma (Fig. 1a, b). Figure 1c illustrates the Kaplan–Meier curves on both OS and RFS for GB, LGG, and LIHC.

### Clinical correlation analysis

**Fig. 2:**
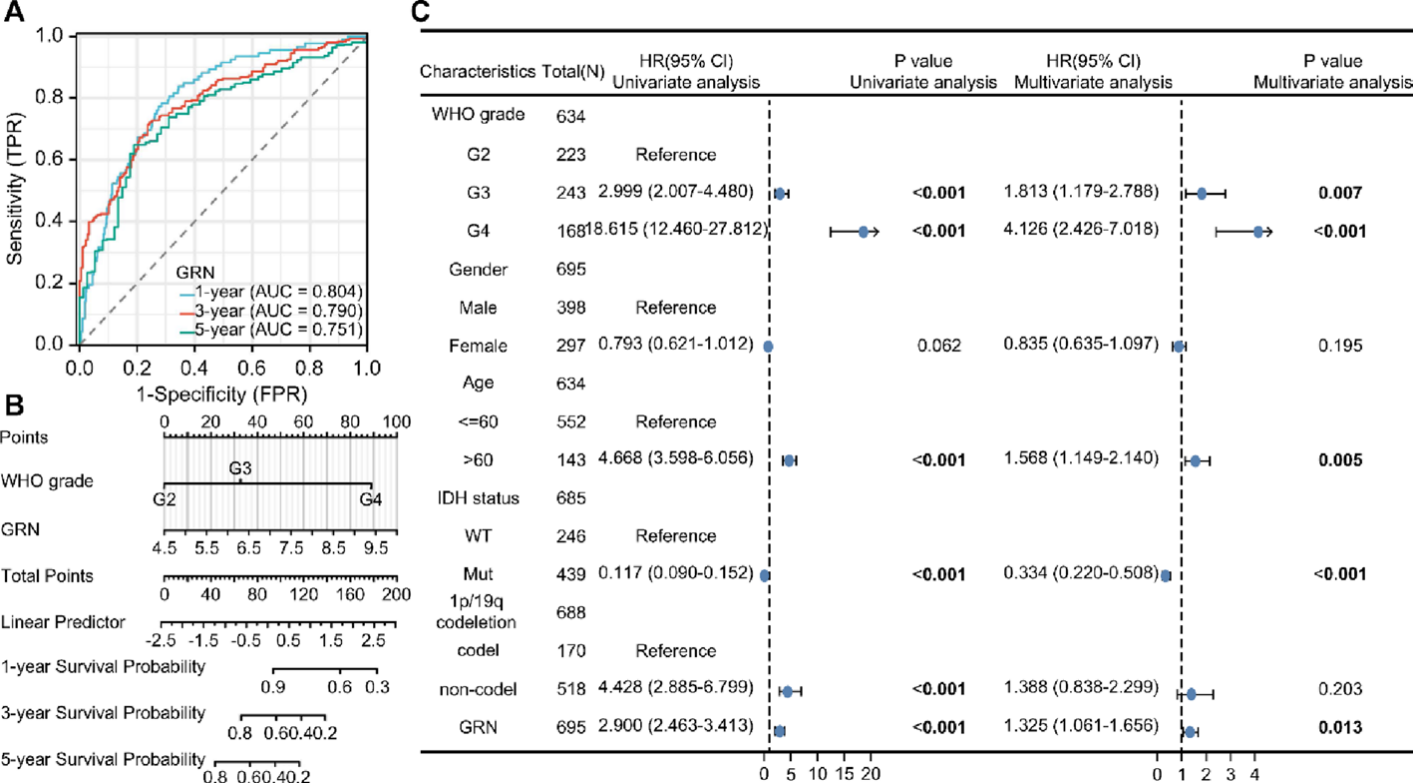
PGRN was an appropriate diagnostic and prognostic marker of glioma, **a** The prognostic value of PGRN in glioma OS through Cox regression. **b** The relationship between PGRN and clinical factors in the prognostic nomogram. **c** The univariate and multivariate Cox regression of PGRN in glioma. Univariate analysis shows significant association between PGRN expression and survival outcomes (Left), multivariate analysis adjusted for relevant clinical covariates, maintaining PGRN’s prognostic significance (Right). PGRN (Progranulin), GRN (gene name of progranulin), OS (Overall Survival)

After differential expression and survival analyses, we found that PGRN may play a role in glioma and LIHC. The diagnostic ROC analysis of PGRN showed that the area under the curve (AUC) of GB, LGG, and LIHC exceeded 0.9 (Fig. 1d). The baseline characteristics of patients with glioma showed high PGRN levels in older adult patients, those with non-1p/19q codel, wild-type isocitrate dehydrogenase (IDH) status, and high-grade tumors (Online Resource 1: Table S3). No significant differences were observed in the baseline characteristics of patients with LIHC between the two groups (Online Resource 1: Table S4). Further Cox regression analysis of glioma OS revealed AUCs of 0.804, 0.790, and 0.751 at 1, 3, and 5 years, respectively (Fig. 2a). The prognostic nomogram showed a relationship between PGRN levels and clinical factors (Fig. 2b). Univariate and multivariate Cox regression analyses indicated that PGRN is an independent prognostic marker for gliomas (Fig. 2c). These results suggest that PGRN is a reliable diagnostic and prognostic marker for gliomas.

### PGRN promoted the proliferation, migration, and invasion of GL261 cells

**Fig. 3:**
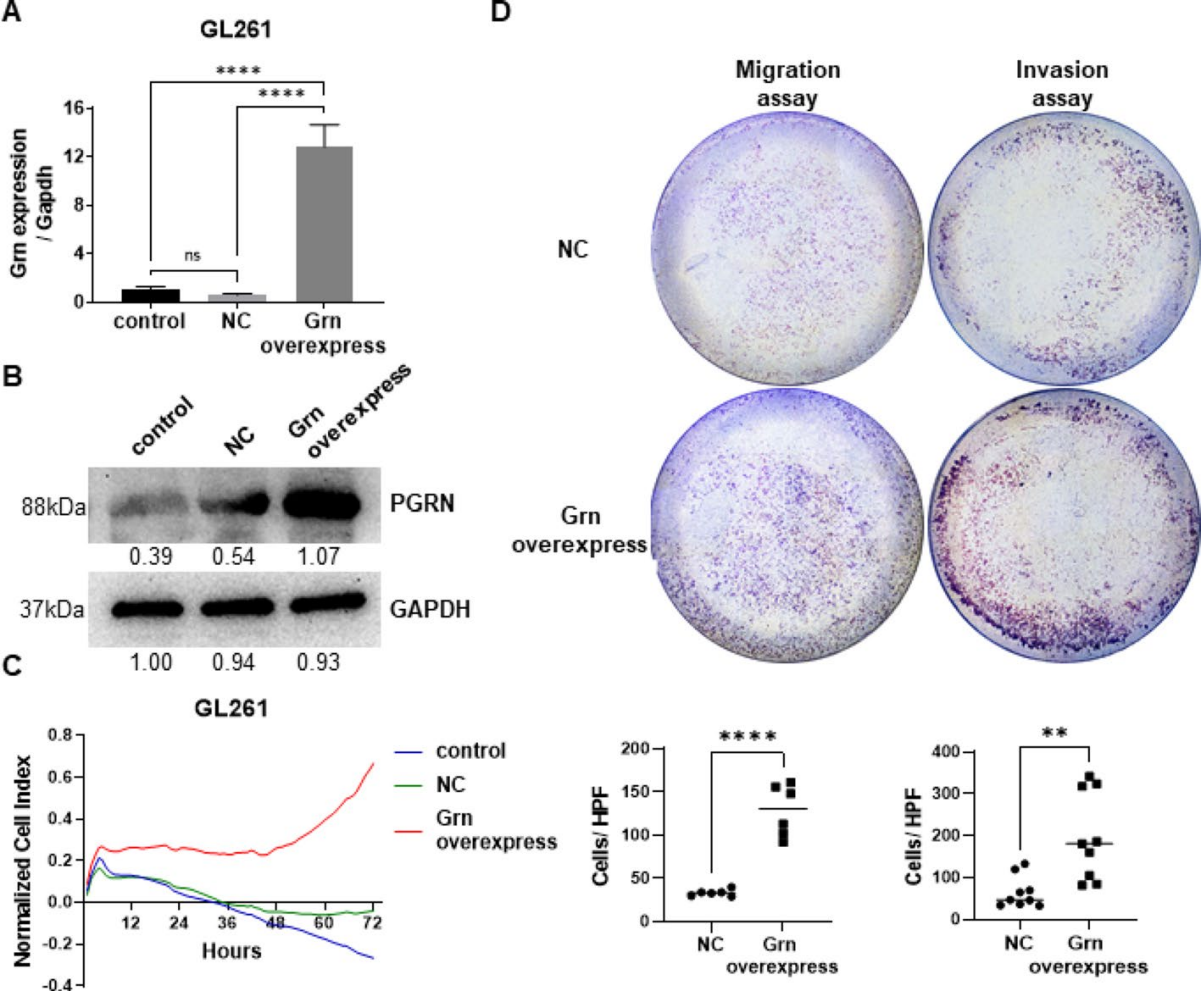
PGRN promoted the proliferation, migration, and invasion of GL261 cells. **a** qPCR analysis confirming successful overexpression of PGRN mRNA in GL261 cells transfected with lentivirus carrying PGRN compared to NC. Relative PGRN expression was normalized to GAPDH. **b** Immunoblotting assay validating PGRN protein overexpression in GL261 cells transfected with lentivirus carrying PGRN compared to NC. GAPDH served as loading control. **c** RTCA demonstrating significantly enhanced proliferation in PGRN-overexpressing GL261 cells compared to NC over a 72-hour monitoring period. **d** Transwell migration and Matrigel invasion assays revealing increased migratory and invasive capabilities of PGRN-overexpressing GL261 cells compared to NC. Representative images (upper) and quantitative analysis (lower) of penetrated cells are shown. Data are presented as means ± SDs, *p < 0.05, **p < 0.01, ****p < 0.0001. PGRN (Progranulin), GRN (gene name of PGRN), NC (Negative Control), RTCA (Real-time cell analysis), CI (cell index)

To investigate the effect of PGRN on glioma, lentivirus carrying either NC or full-length PGRN was transfected into GL261 cells. qPCR assay showed a significant increase in PGRN expression in the overexpression group (Fig. 3a). Immunoblotting confirmed successful transfection (Fig. 3b). RTCA measurements of cell proliferation revealed CI values in the PGRN overexpression group than those in the NC group (Fig. 3c), suggesting that PGRN overexpression promotes glioma cell proliferation. Transwell experiments assessing cell migration and invasion showed increased cell passage through the membrane in the PGRN overexpression group for both migration (Fig. 3d, upper) and invasion (Fig. 3d, lower) experiments, indicating PGRN promotion in glioma cell migration and invasion.

### Effector molecule analysis

**Fig. 4:**
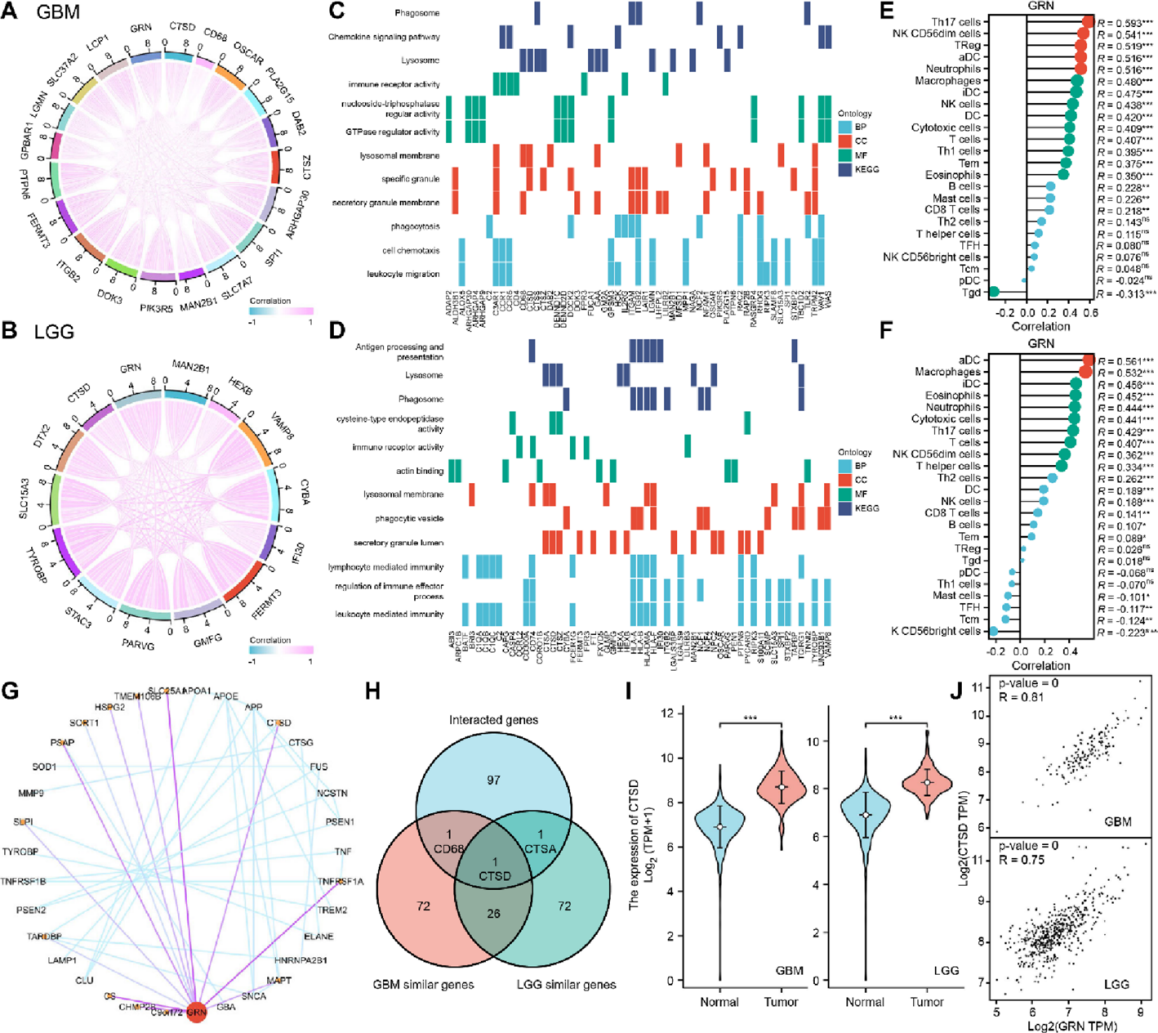
Identification of CTSD as a downstream effector of PGRN in glioma. **a**, **b** The top 100 similar genes in GB and LGG datasets, highlighting potential functional associations. **c**, **d** GO and KEGG enrichment analysis of PGRN-correlated genes in GB and LGG. **e**, **f** ssGSEA algorithm assessed the effect of PGRN on immune cell infiltration characteristics. **g** The interactions between PGRN and genes with high prediction scores. **h** The overlapping genes of the GB-similar, LGG-similar, and interacted gene set. (**i**, **j**) CTSD was highly expressed in tumor tissues and positively correlated with PGRN in GB and LGG. PGRN (Progranulin), CTSD (Cathepsin D), glioblastoma (GB), lower grade glioma (LGG), GO (Gene Ontology), KEGG (Kyoto Encyclopedia of Genes and Genomes), ssGSEA (single sample Gene Set Enrichment Analysis)

Given the tumor-promoting role of PGRN, we investigated its mechanism of action. The top 100 similar genes in GB and LGG were explored using GEPIA 2.0. Figure 4a, b shows genes with a high Pearson’s correlation coefficient, including CTSD, CD68, and MAN2B1. GO and KEGG enrichment analyses indicated that these genes were predominantly involved in inflammation and immune response pathways in both GB and LGG (Fig. 4c, d). The ssGSEA algorithm assessed the effect of PGRN on immune cell infiltration. The results revealed positive correlations with Th 17, NK CD56dim, TReg, aDC (antigen - presenting dendritic cell) cells, and neutrophils in GB, and with aDC cells and macrophages in LGG (Fig. 4e, f). These data suggest that PGRN regulates immune cell function and infiltration into tumor tissues.

STRING analysis identified interactions between PGRN and the genes with high prediction scores (Fig. 4g). Venn analysis revealed overlapping genes among the GB-similar, LGG-similar, and interacting gene sets (Fig. 4h). Based on these findings, we conclude that CTSD acts as an effector molecule of PGRN in gliomas. CTSD is a protease located in the lysosome, cytosol, and extracellular fluid and participates in autophagy [27]. Analysis of the data revealed that CTSD exhibited elevated levels of expression in tumor tissues and demonstrated a positive correlation with PGRN expression in both GB and LGG (Fig. 4i, j).

### PGRN interacted with and promoted CTSD maturation

**Fig. 5:**
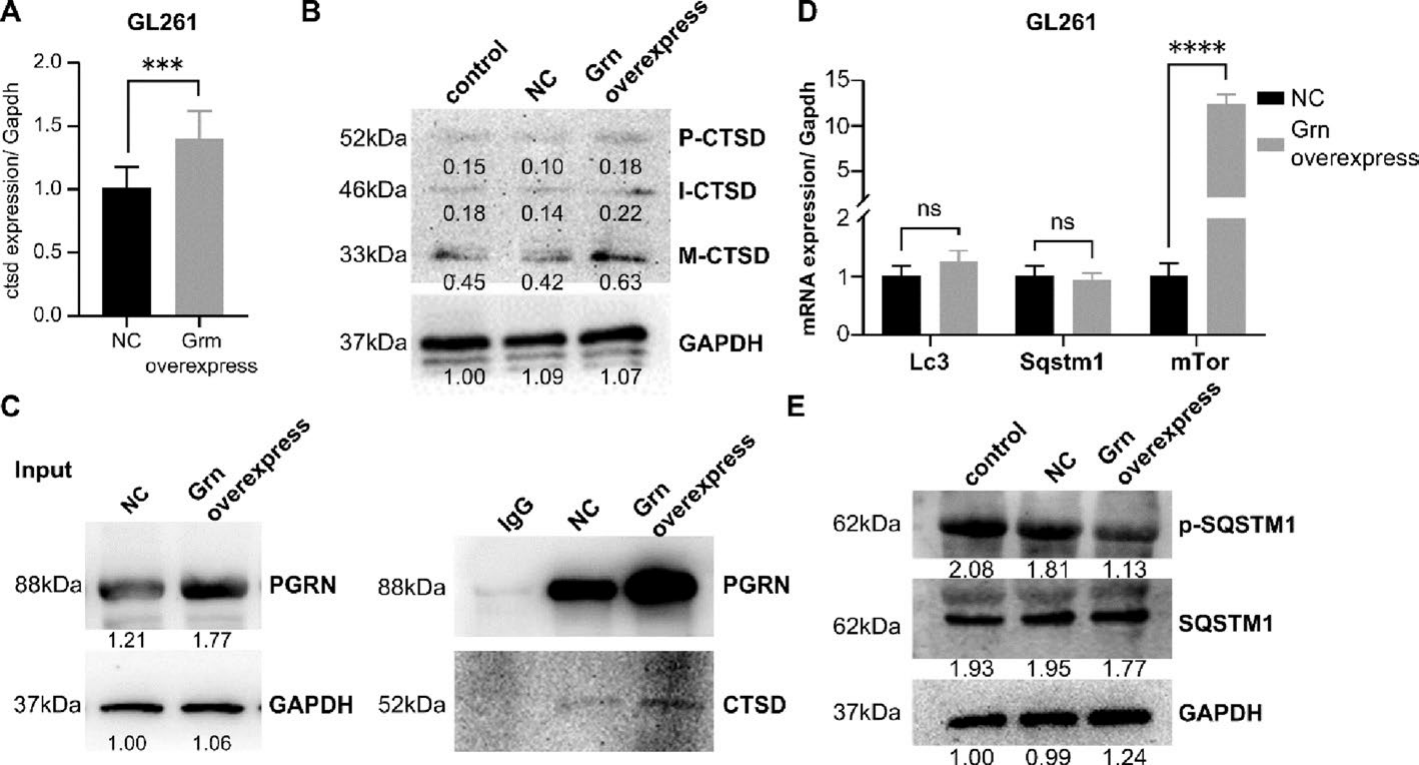
PGRN interacted with and promoted CTSD maturation. **a** qPCR assay showed that PGRN overexpression led to an increase in CTSD mRNA expression. **b** Immunoblotting analysis confirmed that PGRN promoted CTSD maturation. **c** Co-IP showed that the immunoprecipitation of PGRN led to the co-immunoprecipitation of CTSD. **d** qPCR assay showed key autophagy regulators SQSTM1, LC3, and mTOR mRNA levels as indicated. **e** Immunoblotting analysis showed the result after PGRN overexpression as indicated. Data are presented as means ± SDs, *p < 0.05, **p < 0.01, ****p < 0.0001. PGRN (Progranulin), CTSD (Cathepsin D), qPCR (quantitative Real-time PCR), Co-IP (co-immunoprecipitation), SQSTM1 (sequestosome 1, also names p62), LC3 (microtubule-associated protein 1 A/1B-light chain 3), and mTOR (mechanistic target of rapamycin)

QPCR assay showed that PGRN overexpression increased CTSD mRNA expression (Fig. 5a). Immunoblotting analysis confirmed that PGRN increased mature 33-kDa fragments without affecting the levels of intermediate 46-kDa and immature 52-kDa fragments of CTSD, indicating that PGRN promoted CTSD maturation (Fig. 5b).

To determine whether PGRN forms a complex with CTSD, co-IP was performed using GL261 cells. These findings indicate that the immunoprecipitation of PGRN resulted in the co-immunoprecipitation of CTSD. Moreover, PGRN overexpression enhanced the co-immunoprecipitation of CTSD (Fig. 5c).

### PGRN promoted autophagy of GL261 cells

Given the established role of CTSD in autophagy, we investigated the effect of PGRN on autophagy. The classic autophagic molecule sequestosome 1 (SQSTM1), autophagy-related protein LC3 (LC3), and mammalian target of rapamycin were detected. QPCR assay showed that SQSTM1 and LC3 mRNA levels remained unchanged following PGRN overexpression (Fig. 5d). Although SQSTM1 expression remained unchanged, its phosphorylation decreased in response to PGRN overexpression (Fig. 5e).

### CTSD is an effector for the PGRN function

**Fig. 6:**
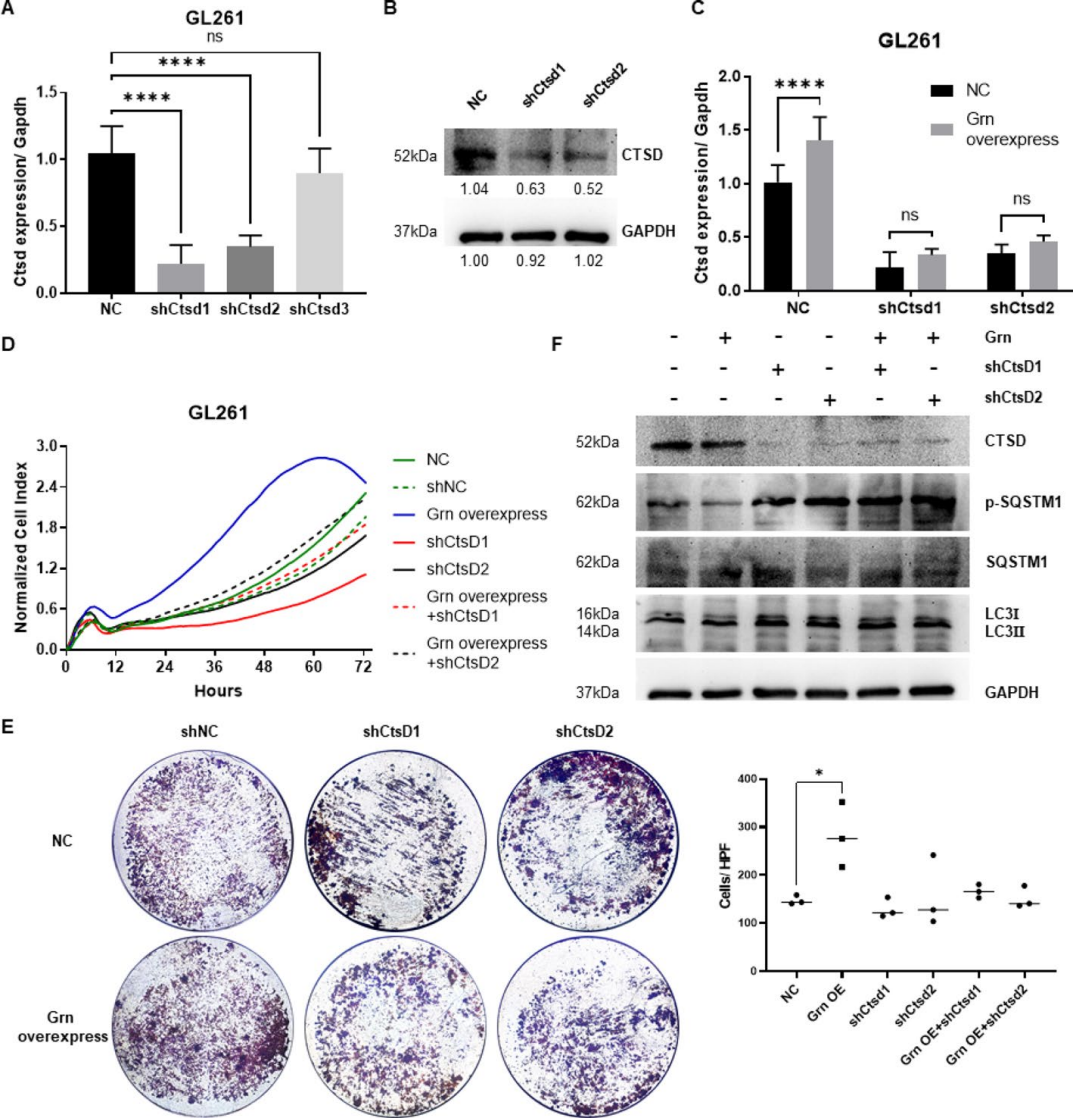
CTSD silencing with shCTSD rescued the effect of PGRN (**a**–**c**) qPCR and immunoblot assay proved the effectiveness of shCTSD1. (**d**,** e**) RTCA curves and invasion assay indicated that shCTSD1 inhibited the proliferation-promoting ability of PGRN. (**f**) Immunoblotting assay confirmed that PGRN increased LC3-II levels but decreased SQSTM1 phosphorylation, whereas shCTSD increased both LC3-II levels and SQSTM1 phosphorylation. Data are presented as means ± SDs, *p < 0.05, **p < 0.01, ****p < 0.0001. PGRN (Progranulin), CTSD (Cathepsin D), shCTSD (short hairpin RNA CTSD), qPCR (quantitative Real-time PCR), RTCA (Real-time cell analysis), SQSTM1 (sequestosome 1, also names p62), LC3 (microtubule-associated protein 1 A/1B-light chain 3).

To investigate the role of CTSD in PGRN regulating GL261 cells, short hairpin (sh) CTSD plasmids were constructed and transfected into GL261 cells. qPCR and immunoblot assays demonstrated the efficacy of shCTSD1 (Fig. 6a–c). The RTCA curves demonstrated that the proliferation rate in the PGRN overexpression group overpassed that of the NC group, whereas the shCTSD1 group exhibited opposite outcomes. Additionally, no difference was observed between the NC and PGRN overexpression + shCtsd1 groups, indicating that shCTSD1 inhibited the proliferation-promoting ability of PGRN (Fig. 6d). The invasion assay showed similar results; cell invasion was promoted by PGRN but inhibited by shCTSD1, with shCTSD1 rescuing the effect of PGRN (Fig. 6e). Regulation of autophagy by CTSD was also examined. Immunoblotting confirmed that PGRN increased LC3-II levels but decreased SQSTM1 phosphorylation, indicating its autophagy-promoting effect. In contrast, shCTSD increased both LC3-II levels and SQSTM1 phosphorylation, potentially reversing the effects of PGRN (Fig. 6f).

## Discussion

This study focused on PGRN and identified it as a promising indicator for glioma diagnosis and prognosis. Bioinformatic analysis showed that PGRN was highly expressed in LGG, GB, and LIHC and was significantly negatively correlated with survival time. Analysis of baseline characteristics indicated that PGRN correlated with the World Health Organization grade, IDH status, and 1p/19q co-deletion in glioma, but not with the TNM stages of LIHC. Subsequent analyses suggested that PGRN exhibited high diagnostic and prognostic scores and was an independent prognostic factor for gliomas. These findings align with previous research indicating that GRN could serve as a valuable diagnostic and prognostic indicator and could potentially play a crucial role in the development and progression of gliomas [28].

Our experimental findings further confirmed that PGRN promotes the proliferation, migration, and invasion of GL261 cells. The results are consistent with those of previous studies indicating that PGRN overexpression facilitates cellular proliferation, whereas PGRN silencing hampers this process in GB S1R1 cells. Cells with silenced PGRN exhibit a distinct G2/M phase arrest when exposed to 250 µM Temozolomide (TMZ), while PGRN overexpression effectively mitigates the G2/M arrest induced by TMZ in PGRN-silenced cells [11]. PGRN not only plays a role in the glioma parenchyma but also has an effect on the microenvironment. PGRN is highly expressed in tumor-associated macrophages (TAM) in the GB environment. Treatment using supernatants derived from bone marrow-derived macrophages (BMDM) of Grn-/- mice reduces GB cell proliferation and protein expression associated with the cell cycle and mesenchymal subtype. Moreover, Grn-/- BMDM supernatant treatment decreases phosphorylated STAT3 levels in GB cells and decreased IL-6 and IL-10 expression in Grn-/- BMDMs [29]. Overall, the data analysis and experimental results suggested that PGRN contributes to the stimulation of tumor growth throughout the progression of glioma. Additionally, it may serve as a useful diagnostic and prognostic biomarker.

The following bioinformatic analysis revealed that PGRN expression correlated with immune cell infiltration in glioma, including macrophages and aDCs. This suggested a regulating role for PGRN in modulating the tumor immune microenvironment. Emerging evidence shows that mitochondrial metabolic reprogramming centrally controls the functional state of these immune cells. Specifically, the immunosuppressive M2 phenotype of TAMs is associated with enhanced mitochondrial oxidative phosphorylation [30]. Similarly, aDCs are regulated by mitochondrial function and metabolic processes [31], suggesting that the association between PGRN and immune infiltration may stem from its potential influence on mitochondrial reprogramming in TAMs and aDCs.

Herein, we explored the mechanisms underlying the role of PGRN in gliomas. Based on the analysis of PGRN-related genes, CTSD is considered an effector molecule. CTSD is a protease synthesized in the rough endoplasmic reticulum (ER). After removing the signal peptide, a 52-kDa pro-CTSD was transferred to lysosomes, endosomes, and phagosomes. Pro-CTSD may be cleaved into a 48-kDa intermediate fragment and finally processed into mature CTSD in the lysosome. As a glycoprotein, pro-CTSD is secreted into the extracellular fluid, taken up by other cells, and cleaved by cancer cells and fibroblasts [32].

CTSD involves multiple physiological and pathological processes. A bioinformatic analysis that used machine learning to create an optimal prognostic index for survival-related variables in patients with GB identified CTSD as a significant gene [33]. As a gene closely associated with the clinical malignancy and prognosis of gliomas, CTSD is elevated in radioresistant GB U251 clones, while its silencing enhances radiosensitivity. This highlights the crucial role of CTSD in GB radiosensitivity, making it a potential biomarker and therapeutic target [34]. In the present study, CTSD exhibited elevated expression levels in tumor tissues and demonstrated a positive correlation with PGRN expression in both GB and LGG, which is consistent with the above studies.

Gene detection and GO/KEGG enrichment analyses show that PGRN is primarily associated with phagosomes, lysosomes, inflammation, and immunity. CTSD mainly functions in lysosomes, where it hydrolyzes prosaposin into lysosomal activator proteins known as saposins A–D [35]. Similar to PGRN, loss of function in CTSD is associated with lysosomal storage disorders and neurodegeneration [36]. Several studies have reported that PGRN, particularly its cleavage product GRNE, interacts with pro-CTSD to promote CTSD maturation, while PGRN deficiency reduces CTSD activity [35, 37–39]. Our data confirm these findings in gliomas and show that CTSD is crucial in PGRN regulation, as CTSD knockdown reverses PGRN’s effect on glioma cell invasion and proliferation.

As a lysosomal protease, CTSD participates directly in autophagy. Previous reports indicate that radioresistant GB U251 cells exhibit elevated autophagy [34]. Suppression of CTSD increases autophagosome formation but decreases autolysosome formation, ultimately reducing autophagy and enhancing radiosensitivity [34]. LC3 and SQSTM1 are essential proteins involved in the regulation and execution of autophagy. LC3 conversion from LC3-I to LC3-II indicates autophagosome formation. SQSTM1 serves as both a receptor for LC3 and its substrate. Its levels are inversely related to autophagic flux: low when flux is efficient and high when it is impaired. Therefore, monitoring these proteins effectively assesses autophagic activity [40–41]. In this study, PGRN promoted CTSD maturation and LC3-II accumulation, while inhibiting SQSTM1 phosphorylation, indicating its autophagic role in glioma. Conversely, CTSD knockdown exhibited the opposite effect by increasing both LC3-II levels and SQSTM1 phosphorylation, indicating autophagy inhibition.

The PGRN-CTSD axis may extend beyond lysosomal autophagy to involve mitochondrial-lysosomal crosstalk. Mitochondrial stress pathways, such as those involving PTEN-induced putative kinase 1 (PINK1), affect lysosomal activity and autophagy. Recent studies demonstrate that PINK1 insufficiency can be therapeutically exploited in gastric cancer. Such drug combinations inducing mitochondrial pathology selectively activate apoptosis in cancer cells while sparing normal tissues [42]. This suggests potential synergy between mitochondrial-targeting agents and strategies that modulate the PGRN–CTSD autophagy axis.

However, the role of PGRN in autophagy remains controversial, with conflicting evidence reported in the literature: PGRN deficiency alleviates autophagy-dependent MHC class I degradation and leads to LC3-II and SQSTM1 accumulation, attenuating the autophagic response [43–45]; conversely, PGRN-deficient neurons showed increased autophagy and enlarged lysosomes [46]. Moreover, the role of autophagy in tumorigenesis is also controversial. It acts as a survival pathway in early tumorigenesis but promotes tumor aggressiveness at later stages [47]. Considering that GL261 is derived from GB, a high-grade glioma, its autophagic characteristics align with late-stage tumors.

This study provides detailed mechanistic evidence for the PGRN-CTSD axis in glioma progression. However, these key functional and biochemical validations were primarily performed in the GL261 murine cell model. Although PGRN’s oncogenic role is conserved across species and cell lines, future studies using a panel of human glioma cells and patient-derived glioblastoma cell lines are essential to fully confirm its universal therapeutic potential across diverse glioma subtypes and patient populations.

## Conclusions

Overall, our study identified PGRN as a promising biomarker for glioma diagnosis and prognosis. These findings indicate that PGRN enhances glioma cell proliferation, migration, invasion, and autophagy by interaction with and upregulation of CTSD, thereby enhancing our understanding of glioma diagnosis and prognosis.

## Supplementary Information

Below is the link to the electronic supplementary material.

Supplementary Material 1.

## Data Availability

All data generated or analyzed during this study are included in this published article and its supplementary information files. Data used for bioinformatics analysis are sourced from the datasets stored in the Xiantao Academic Platform ( https://www.xiantaozi.com/ ) and the Gene Expression Profiling Interactive Analysis (GEPIA) platform ( http://gepia2.cancer-pku.cn/#index ).
