# Supplementary material for: Progranulin promotes glioma progression via interaction with cathepsin D and serves as a diagnostic and prognostic biomarker
Source: Discov Oncol. 2026 Jan 27;17:336. doi: 10.1007/s12672-026-04529-9 (PMC12917043; doi:10.1007/s12672-026-04529-9)
Supplement: Supplementary file 1 — Supplementary Material 1. [file 12672_2026_4529_MOESM1_ESM.docx]

*Discover Oncology*

**Supplementary Information**

**Progranulin Promotes Glioma Progression via Interaction with Cathepsin D and Serves as a Diagnostic and Prognostic Biomarker**

Chunming Zhao^1,2†^, Jiamei Guo^2,3†^, Zhong Zhou^2,4^, Xulong Huang^2,4^, Yifan Hai^5^, Wenbo Gao^5^, Chaohang Chen^2,4^, Guokai Dong^2,4^, Hongxing Cai^2,4^, Shanshan Li^2,4^*

^1^Department of Human Anatomy, Xuzhou Medical University, Xuzhou, Jiangsu, China

^2^Jiangsu Medical Engineering Research Center of Gene Detection, Xuzhou, Jiangsu, China

^3^Judicial Expertise Office, Jiaxing University, Xuzhou, China

^4^Department of Forensic Medicine, Xuzhou Medical University, Xuzhou, Jiangsu, China

^5^The First Clinical Medical College, Xuzhou Medical University, Xuzhou, Jiangsu, China

^†^These authors contributed equally to this work.

***Corresponding author:** Shanshan Li

ORCiD: 0000-0001-6443-958X

Email: [sharon6128126@xzhmu.edu.cn](mailto:sharon6128126@xzhmu.edu.cn)

**Table S1**. Primer sequences used in this study

| Gene name | Sense (5'-3') | Antisense (5'-3') |
| --- | --- | --- |
| CtsD | CGCAGTGTTTCACAGTCGTC | TCAAAGACCGGAAGCACGTT |
| Lc3 | TTGGTCAAGATCATCCGGCG | TTCTTGGGAGGCGTAGACCA |
| Sqstm1 | GATAGCCTTGGAGTCGGTGG | TCAGCCTCTGTAGATGGGTCC |
| Grn | ACCCTCTTCTGGACACATGG | GGAAGTCCCAGACACAGTGA |
| mTor | CCGCCTTCACAGATACCCAG | AGGGATGCCAAGACACAGTAG |

**Table S2**. Antibodies used in this study

| **Antibody** | **RRID** |
| --- | --- |
| CTSD | (Santa Cruz Biotechnology Cat# sc-377124, RRID:AB_2890647) 1:500 |
| SQSTM1 | (Santa Cruz Biotechnology Cat# sc-48402, RRID:AB_2255371) 1:1000 |
| GAPDH | (Santa Cruz Biotechnology Cat# sc-365062, RRID:AB_10847862) 1:1000 |
| P-SQSTM1 | (Proteintech Cat# 29503-1-AP, RRID:AB_2923592) 1:2000 |
| PGRN | (Abcam Cat# ab187070, RRID:AB_2885106) 1:1000 |
| Peroxidase AffiniPure Goat Anti-Mouse IgG (H+L) | (Jackson ImmunoResearch Labs Cat# 115-035-003, RRID:AB_10015289) 1:10000 |
| Peroxidase AffiniPure Goat Anti-Rabbit IgG (H+L) | (Jackson ImmunoResearch Labs Cat# 111-035-003, RRID:AB_2313567) 1:10000 |
| HRP Conjugated Anti-mouse IgG for IP Nano-secondary antibody | (Huabio Cat# NBI02H, RRID:AB_3668838) 1:5000 |

CTSD, cathepsin D; SQSTM1, sequestosome 1; GAPDH, glyceraldehyde-3-phosphate dehydrogenase; PGRN, progranulin; IgG, immunoglobulin G

**Table S3**. Baseline data of patients with glioma (LGG+GBM)

| Characteristics | Low expression of GRN | High expression of GRN | P value |
| --- | --- | --- | --- |
| n | 349 | 350 |  |
| WHO grade, n (%) |  |  | <0.001 |
| G2 | 165 (25.9%) | 59 (9.3%) |  |
| G3 | 131 (20.6%) | 114 (17.9%) |  |
| G4 | 17 (2.7%) | 151 (23.7%) |  |
| IDH status, n (%) |  |  | <0.001 |
| WT | 45 (6.5%) | 201 (29.2%) |  |
| Mut | 299 (43.4%) | 144 (20.9%) |  |
| 1p/19q codeletion, n (%) |  |  | <0.001 |
| Non-codel | 219 (31.6%) | 301 (43.5%) |  |
| Codel | 129 (18.6%) | 43 (6.2%) |  |
| Age, median (IQR*) | 40 (33, 52) | 52 (36, 62) | <0.001 |

*IQR: minimum and maximum ages of included individuals.

The patients were divided into high and low groups by PGRN median value. LGG, low-grade glioma; GBM, glioblastoma; PGRN, progranulin

**Table S4**. Baseline data of patients with LIHC

| Characteristics | Low expression of GRN | High expression of GRN | P value |
| --- | --- | --- | --- |
| n | 187 | 187 |  |
| Pathologic T stage, n (%) |  |  | 0.145 |
| T1 | 100 (27%) | 83 (22.4%) |  |
| T2 | 41 (11.1%) | 54 (14.6%) |  |
| T3&T4 | 43 (11.6%) | 50 (13.5%) |  |
| Pathologic N stage, n (%) |  |  | 1.000 |
| N0 | 128 (49.6%) | 126 (48.8%) |  |
| N1 | 2 (0.8%) | 2 (0.8%) |  |
| Pathologic M stage, n (%) |  |  | 1.000 |
| M0 | 135 (49.6%) | 133 (48.9%) |  |
| M1 | 2 (0.7%) | 2 (0.7%) |  |
| Age, median (IQR*) | 62 (52, 69) | 61 (51, 68) | 0.353 |

*IQR: minimum and maximum ages of included individuals.

The patients were divided into high and low groups by PGRN median value. LIHC, liver hepatocellular carcinoma; PGRN, progranulin


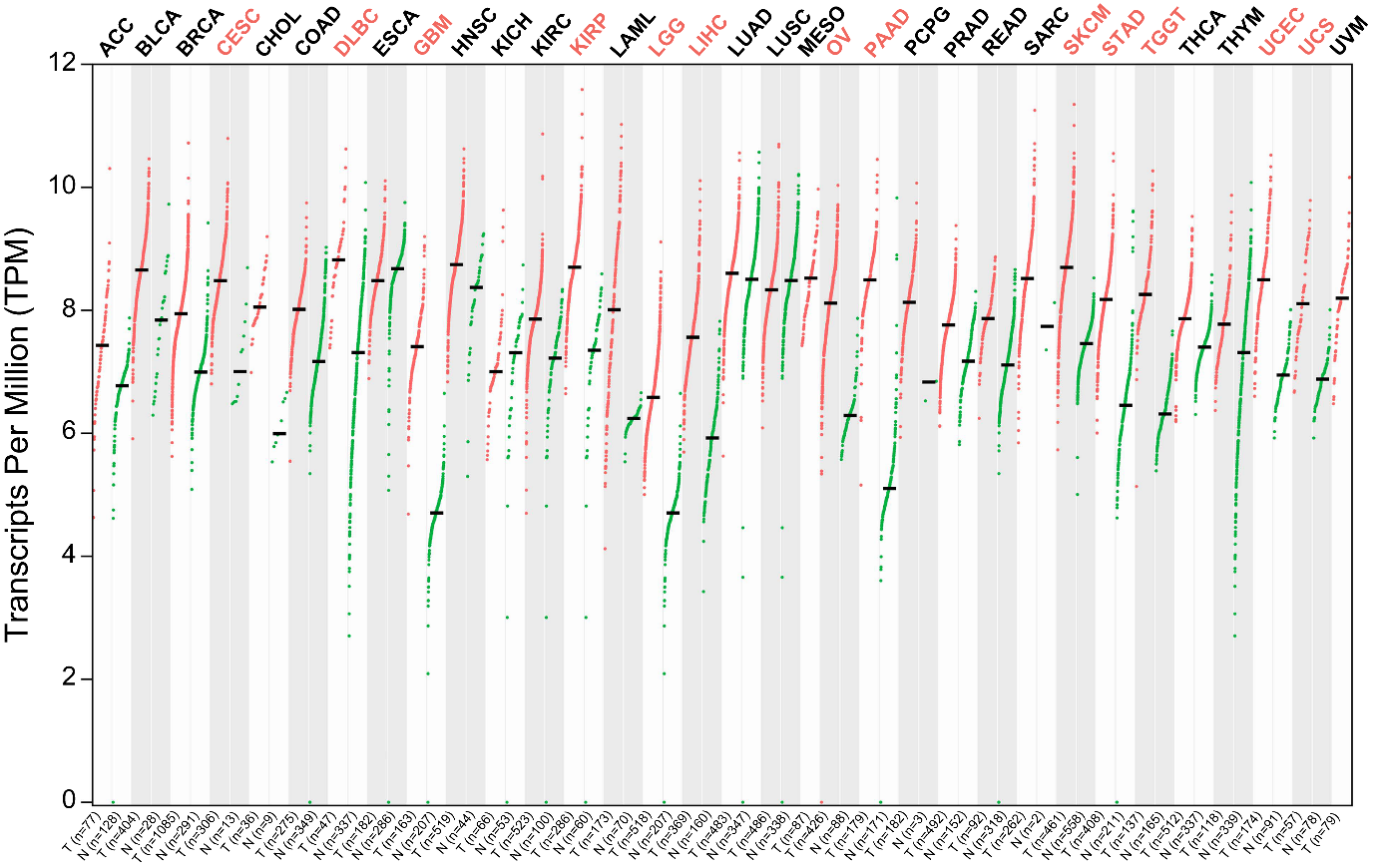


**Fig. S1** The mRNA expression of PGRN in 33 types of tumors compared with matched normal tissues. The picture was formed by GEPIA 2.0, a web-based tool that analyses RNA sequencing expression data from publicly available datasets of TCGA and GTEx projects. The datasets can be downloaded from UCSC XENA (https://xenabrowser.net/datapages/). TCGA provides comprehensive genomic data for various cancers, while GTEx offers expression data for normal tissues across multiple organs. In GEPIA 2.0, RNA sequencing expression data from 9,736 tumors and 8,587 normal samples is processed and normalized using a standard pipeline. Transcripts Per Million represent the mRNA level of PGRN in various tumors
